# Supplementary material for: Characterization and Evaluation of Layered Bi2WO6 Nanosheets as a New Antibacterial Agent
Source: Antibiotics (Basel). 2021 Sep 3;10(9):1068. doi: 10.3390/antibiotics10091068 (PMC8468918; doi:10.3390/antibiotics10091068)
Supplement: Supplementary file 1 [file antibiotics-10-01068-s001.zip › antibiotics-1323914-supplementary.pdf]

SUPPLEMENTARY MATERIAL

METHODX

Synthesis

The synthesis of Bi_2WO_6 monolayers was performed by a hydrothermal method, as described previously [4]. Sodium tungstate dihydrate [$Na_2WO_4.2H_2O$] (≥99%, Sigma-Aldrich), bismuth nitrate pentahydrate [$Bi(NO_3)_3.5H_2O$] (≥98.0%, Sigma-Aldrich) and hexadecyltrimethylammonium – CTAB [$CH_3(CH_2)_{15}N(Br)(CH_3)_3$] were used as starting precursors. In a synthesis procedure, 1 mmol of $Na_2WO_4.2H_2O$, 2 mmol of $Bi(NO_3)_3.5H_2O$ and 0.05 g of CTAB were dissolved in 80 mL of deionized water. This aqueous solution was stirred by 30 min at an average speed of 1500 rpm. The resulting solution was transferred to a 100 mL Teflon-lined stainless-steel autoclave and maintained at 120 °C for 24 h. The white precipitates were repeatedly washed with deionized water and dried in an air oven at 60 °C for 10 h.

Structural characterization

Structural characterization was performed by X-ray diffraction (XRD) using a Mini-Flex Rigaku diffractometer equipped with a Cu Kα (λ = 1.5418 Å) radiation in the 2θ range of 10°–80°, with a step size of 0.02° and a count time of 2 s/step. The morphological analysis of the Bi_2WO_6 monolayers was performed in a scanning electron microscope (SEM) model Vega3 Tescan. Atomic force microscopy images were recorded using NTMDT microscope in ambient conditions (25 °C, 1 atm). AFM images were acquired using a 0.5 Hz scanning frequency in intermittent contact mode. The sample was dispersed in ethanol using an ultrasonic bath. A drop of the diluted dispersion was deposited onto a glass substrate and dried in air. Fourier Transform Infrared (FT-IR) spectra were obtained in the range from 450 to 4000 cm^{-1} using KBr pellets as a reference using a Perkin Elmer Spectrum Two spectrophotometer in transmittance mode. Raman measurements were performed using a Horiba LabRaman spectrometer equipped with a liquid N_2-cooled CCD system. The 633 nm line laser was used to excite the Raman signal. An Olympus microscope lens with a focal distance of 20.5 mm and a numerical aperture of 0.35 was used to focus the laser beam on the sample surface. The slit was set to a resolution of 2 cm^{-1}. The spectrum was obtained using four accumulations and an acquisition time of 60 s. The laser power on the sample surface was kept lower than 5 mW to avoid local heating effects.

Analysis of antibacterial activity

The standard bacterial strains (*E. coli* ATCC 25922 and *S. aureus* ATCC 25923) and multidrug-resistant (*S. aureus* 10 and *E. coli* 06) were provided by the Laboratory of Microbiology and Molecular Biology (LMBM) from the Regional University of Cariri (URCA). The antibiotics amikacin and gentamicin, as well as all reagents used in the tests, were purchased from Sigma Chemical Co. (St. Louis, USA). All drugs were diluted in distilled water to an initial concentration of 1024 μg/mL.

The MIC of each drug was determined using the broth microdilution method. All strains were cultured in Heart Infusion Agar (HIA) solid medium for 24 h at 37 °C. Samples were transferred from the solid medium to test tubes containing sterile saline solution and turbidity was assessed using a value of 0.5 on the McFarland scale. Each inoculum was prepared with 10 % BHI at a ratio of 1:9. Then, 100 μL of inoculum in the medium was transferred to wells in a 96-well plate with 100 μL of the drugs in concentrations ranging from 512 to 8 μg/mL, followed by incubation at 37 °C for 24 h. Wells containing only the inoculum in BHI were used as growth control. After incubation, 20 μL of a 0,01% Sodium resazurin solution was added to each well, followed by incubation for 1 h at room temperature. A change in color of the solution from blue to red indicated bacterial growth. MIC was defined as the lowest concentration capable of inhibiting bacterial growth. All experiments were carried out in triplicate.

Analysis of antibiotic resistance modulation by Bi_2WO_6 monolayers

The antibiotic-enhancing activity was evaluated using the methodology described by Coutinho et al. [11]. To this end, the bacterial inoculant was prepared in BHI as described above, and Bi_2WO_6 monolayers were added at a subinhibitory concentration (equivalent to its MIC÷8). The wells in a 96-well plate were filled with 100 μL of this solution, followed by the addition of 100 μL of each antibiotic in concentrations ranging from 512 to 0.5 μg/mL. The MIC of each drug in the presence or absence of Bi_2WO_6 monolayers was determined, and the occurrence of synergism was interpreted as increased antibiotic activity. Experimental controls and readings were performed as previously described.

Evaluation of antibiotic-modulating activity in association with LED light exposure

The Light Emitting Diodes-LED device (a light emitting diode; NEW Estética®) was used in the experimental protocols. This device has red, blue and yellow light spectra and allows the combination of these colors. The LEDs with wavelength pre-determined by the apparatus used were: blue (415 nm), red (620 nm) and yellow (590 nm). To evaluate the effect of LED light exposure on bacterial growth in antibacterial activity modulation, cultures and treatments were carried out as previously described. Plates were exposed to blue, red, or yellow light for 20 min and then incubated at 37 °C for 24 h. Plates without exposure to LED light were used as experimental controls. Readings were performed as described before.

Statistical analysis

Data are expressed as mean ± standard deviation and differences were evaluated through analysis of variance (ANOVA) followed by *Bonferroni's* post-test using the GraphPad Prism 6.0 software. The differences with a $p < 0.05$ were considered significant.
